# Dark-field X-ray ptychography: Towards high-resolution imaging of thick and unstained biological specimens

**DOI:** 10.1038/srep35060

**Published:** 2016-10-13

**Authors:** Akihiro Suzuki, Kei Shimomura, Makoto Hirose, Nicolas Burdet, Yukio Takahashi

**Affiliations:** 1 Graduate School of Engineering, Osaka University, 2-1 Yamada-oka, Suita, Osaka 565-0871, Japan; 2 RIKEN SPring-8 Center, 1-1-1 Kouto, Sayo-cho, Sayo, Hyogo 679-5148, Japan

## Abstract

The phase shift of light or electrons in objects is now necessary for probing weak-phase objects such as unstained biological specimens. Optical microscopy (OM) and transmission electron microscopy (TEM) have been used to observe weak-phase objects. However, conventional OM has low spatial resolution and TEM is limited to thin specimens. Here, we report on the development of dark-field X-ray ptychography, which combines X-ray ptychography and X-ray in-line holography, to observe weak-phase objects with a phase resolution better than 0.01 rad, a spatial resolution better than 15 nm, and a field of view larger than 5 μm. We apply this method to the observation of both the outline and magnetosomes of the magnetotactic bacteria MO-1. Observation of thick samples with high resolution is expected to find broad applications in not only biology but also materials science.

The visualization of fine structures buried within weak-phase objects such as soft biological tissues and polymers is vital to yield new discoveries in life and materials sciences. The phase shift of light and electrons is now necessary for probing such weak-phase objects. Although optical microscopy with a phase plate, also known as Zernike phase-contrast microscopy, is the most popular tool for observing biological specimens, its spatial resolution is classically limited to ∼200 nm, which is about half the illumination wavelength, owing to the diffraction limit of electromagnetic radiation. Recent super-resolution technologies^1^,^2^ such as structured illumination microscopy can break the diffraction limit, and the use of fluorescent labeling as the imaging basis achieves a resolution of a few tens of nanometers. Alternatively, electron microscopy can image unstained biological specimens, such as frozen hydrated macromolecular complexes that play an important role in molecular biology, at resolutions better than a few nm^3^. The phase contrast mechanism is generated either by conventional defocusing of the lens or with a phase plate^4^; however, only slices of less than 200 nm thickness can be studied owing to the low penetration depth. In contrast, hard X-rays can probe a thick specimen owing to their high penetration power. There are a few techniques for X-ray phase-contrast imaging such as free-space propagation and grating interferometry^5^. These techniques have mainly been developed at third-generation synchrotron facilities. Whole cells are routinely imaged at a resolution of ∼50 nm by X-ray phase-contrast microscopy with focusing/imaging optics. However, further improvement of spatial resolution is not expected owing to theoretical factors and manufacturing limitations. Thus, it is still difficult to use these advanced microscopy techniques for nondestructively observation of thick and weak-phase objects at ∼10 nm resolution.

Coherent diffraction imaging^6^ (CDI) can break the limitation on the spatial resolution of lens-based microscopy, which is a lensless imaging technique based on the iterative phasing method. CDI has been demonstrated using electrons^7^ and X-rays^8^, and has led to a breakthrough in high-resolution X-ray imaging at synchrotron facilities. CDI is sensitive to both phase and absorption contrast. In the framework of weak-phase object approximation^9^, CDI is a phase-contrast imaging technique, in which the oversampled coherent diffraction patterns of a sample are measured, and then the phase images of the sample are reconstructed by phase retrieval calculation. At present, the observation of biological specimens is one of the important applications of CDI with X-rays^10^. Ptychography^11^,^12^, which is a scanning CDI method, is the most practical approach to CDI at third-generation synchrotron facilities since it is free from limitations on the sample size. X-ray ptychography has been applied to the observation of biological specimens such as bacteria^13^,^14^, bone^15^, and sponge^16^. X-ray ptychography, in principle, can be applied to the observation of weak-phase objects at ∼10 nm resolution. However, this is experimentally difficult since an extremely large dynamic range of diffraction patterns has to be measured. The performance of X-ray detectors is currently a major limitation in the high-resolution imaging of weak-phase objects by X-ray ptychography. Recently, to overcome this limitation, we have proposed “dark-field X-ray ptychography”^17^, which combines X-ray ptychography and X-ray in-line holography. Dark-field X-ray ptychography allows us to obscure the low-Q region of diffraction patterns using a beamstop since the in-line hologram complements structural information in the low-Q region, resulting in the compression of the dynamic range of intensities of diffraction patterns at the low-Q region, while the signal-to-noise (S/N) ratio of the diffraction pattern at the high-Q region decreases. We have shown by simulation that the dynamic range of intensities of diffraction patterns is decreased by about three orders of magnitude.

Here, in order to improve the spatial resolution of dark-field X-ray ptychography, we employ two ptychographic datasets with and without in-line hologram, and experimentally demonstrate it. Both the in-line holograms and ptychographic diffraction patterns of a 30-nm-thick Ta test object were measured at SPring-8. Both multiple-mode probes and the object were reconstructed from the hologram/diffraction patterns using a newly proposed reconstruction algorithm. The phase image of the object was reconstructed with a phase resolution better than 0.01 rad, a spatial resolution better than 15 nm, and a field of view larger than 5 μm. In addition, we applied this method to the observation of magnetotactic bacteria MO-1 and successfully visualized the magnetosomes buried within the bacteria.

## Results

### Proof-of-principle experiment

**Figure 1:**
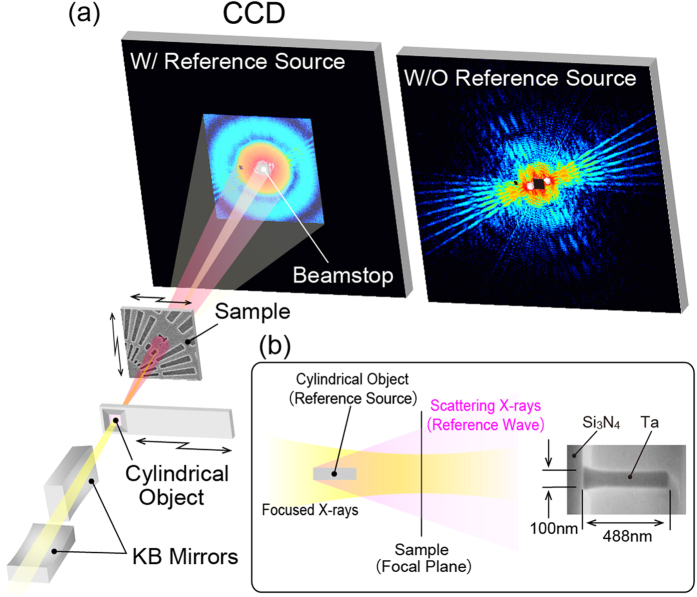
(**a**) Schematic view of experimental setup. A monochromatic X-ray of 6.5 keV is focused by KB mirrors. A cross slit is placed 1 mm upstream of the focusing plane to remove X-ray parasitic scattering from the focusing optics. The sample and the cylindrical object are placed at the focus point and 780 μm upstream from the sample, respectively. A beamstop is positioned immediately before the CCD detector, which is placed 1.2 m downstream from the sample. (**b**) Schematic view of the cylindrical object and its cross-sectional SEM image.

Figure 1(a) shows a schematic view of the experimental setup. The ptychographic measurements were carried out at SPring-8 BL29XUL^18^. A 6.500 keV monochromatic X-ray beam (wavelength λ = 0.1907 nm) was generated by an in-vacuum undulator device and a Si(111) double-crystal monochromator. A slit, which controls the optics for the secondary source, was placed 50 m upstream of Kirkpatrick–Baez (KB) mirror optics. The slit openings in the horizontal and vertical directions were 12 and 16 μm, respectively. The KB mirrors produced a two-dimensionally focused beam with a ∼550 (vertical) × 490 (horizontal) nm^2^ spot size for the full width at half maximum (FWHM), which is close to the diffraction-limited focusing size. The sample was placed at the focus. The flux of the focused X-rays was estimated to be 2.0 × 10^8^ photons/s using a Si PIN photodiode. To remove parasitic X-ray scattering from the KB mirrors, a rectangular slit^19^ was positioned ∼1 mm upstream of the sample position. The key device for dark-field X-ray ptychography is a cylindrical object, which acts as a reference source for in-line holography. The diameter of the cylindrical object should be much less than the above-mentioned diffraction-limited focusing size, while the height should be at least a few hundred nanometers to increase the S/N ratio of in-line holograms. A tantalum cylindrical object was fabricated on a SiN membrane using e-beam lithography technology. A cross-sectional SEM image of the cylindrical object is shown in Fig. 1(b), whose diameter and height were 100 and 488 nm, respectively, which allows it to act as a reference source for an in-line hologram in dark-field X-ray ptychography. The cylindrical object was installed 780 μm upstream of the focus between the rectangular slit and the sample. An in-vacuum front-illuminated CCD detector with a pixel size of 20 × 20 μm^2^ (PyLoN 1300, Princeton Instruments Inc.) was placed 1.219 m downstream of the sample. In the proof-of-principle experiment, a Ta Siemens star chart of 30 nm thickness was used as the sample. The sample was positioned at the focal plane and was mounted on piezoelectric stages inside a high-vacuum chamber that suppressed the air scattering. The sample was illuminated in 7 × 7 overlapping fields of view, which were separated by 300 nm in the horizontal and vertical directions. The diffraction patterns were separately collected at low-Q (−0.023 nm^−1^ ≦ *q*_*x,z*_ ≦ 0.023 nm^−1^) and high-Q (−0.055 nm^−1^ ≦ *q*_*x,z*_ ≦ 0.055 nm^−1^) region to improve the S/N ratio of the high-Q diffraction intensities from the sample^20^. A beamstop of 800 × 800 μm^2^ for the low-Q measurement was placed in front of the CCD detector to block the diffraction patterns in the region −0.0017 nm^−1^ ≦ *q*_*x,z*_ ≦ 0.0017 nm^−1^. For the high-Q measurement, a beamstop of 3000 × 3000 μm^2^ was used. The diffraction patterns in the low-Q region were collected with and without the cylindrical object, respectively. On the other hand, the diffraction patterns in the high-Q region were collected without the cylindrical object. The exposure times at each position were 40 and 400 s for the measurements with and without the cylindrical object, respectively. The total measurement times including readout time from the CCD and positioning error correction were 15.5 h for the measurement with the cylindrical object and 17.0 h for the measurement without the cylindrical object. During the measurement, the temperature change of the apparatus was controlled to less than 0.04 °C over 10 h to suppress thermal drift. The remaining drift was corrected at each scanning position before the measurement by the drift compensation method^21^.

### Diffraction patterns and reconstruction of the test object

**Figure 2:**
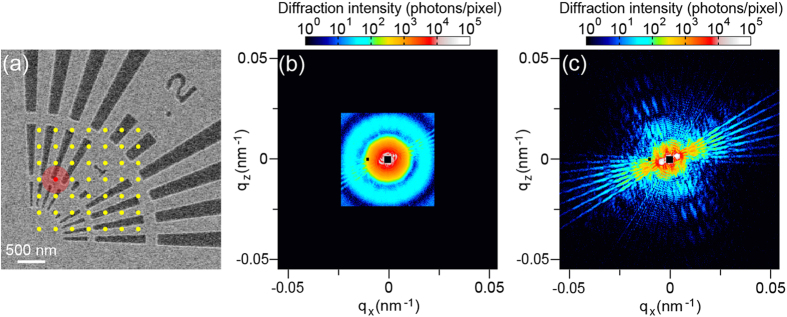
SEM image of the Ta Siemens star test chart. Yellow dots show 7 × 7 illumination points with 300 nm intervals. (**b,c**) Diffraction patterns when the X-ray irradiated the position indicated by the red circle position on the test pattern (**b**) with and (**c**) without the cylindrical object. The maximum spatial frequencies of the diffraction patterns with and without the reference light source are 0.023 and 0.055 nm^−1^, respectively.

**Figure 3:**
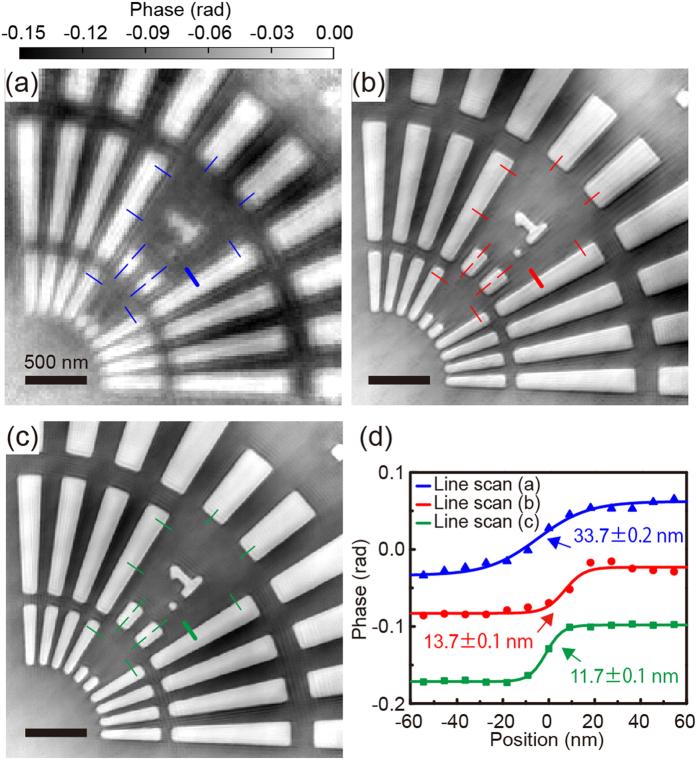
(**a,b**) Phase images of the test patterns reconstructed using a ptychographic dataset (**a**) with and (**b**) without the cylindrical object. (**c**) Phase image reconstructed using both ptychographic datasets. (**d**) Line profiles along blue, red, and green bold lines in reconstructed images.

Figure 2(a) shows a SEM image of the sample. Figure 2(b,c) show the coherent diffraction patterns with and without the cylindrical object, respectively. The diffraction pattern in Fig. 2(c) was created by merging the low-Q and high-Q diffraction patterns. The X-ray irradiation position is indicated by a red dot in Fig. 2(a). In Fig. 2(b), the hologram pattern can be observed, although the circular pattern due to the cylindrical object, *i.e*., the reference beam, predominantly appears. On the other hand, high-Q diffraction patterns from the test sample can be observed in Fig. 2(c). Both the probe and object, for the low-Q dataset with the cylindrical object and the merged dataset without the cylindrical object, were reconstructed using the ePIE algorithm extended to multiple probe modes^22^,^23^. The number of the mode was increased by 1 every 2000 iterations. The iterative process was continued for up to 2000 iterations until that object image could no longer be improved. Six and three probed modes were populated in the reconstruction from the diffraction patterns with and without the cylindrical object, respectively. Figure 3(a,b) show phase images of the test patterns reconstructed from the diffraction patterns with and without the cylindrical object, respectively. The spatial resolution of the reconstructed image in Fig. 3(b) is better than that in Fig. 3(a) owing to the difference in the maximum spatial frequency. However, the image in Fig. 3(b) includes long-period artifacts, particularly seen around number “1”, owing to the loss of information at the low spatial frequency. On the other hand, there are few long-period artifacts in the image in Fig. 3(a) since the in-line hologram complements structural information in the low-Q region although there are still long-period artifacts outside the image due to insufficiency of the overlap constraint.

**Figure 4:**
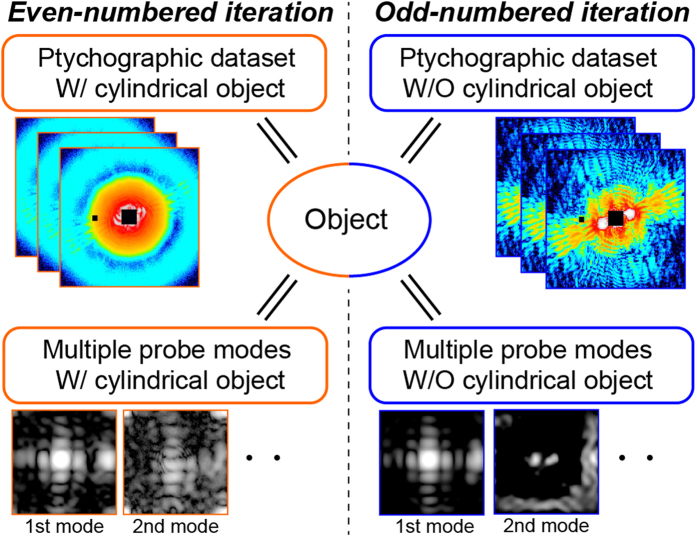
Schematic diagram of the phasing method using two ptychographic datasets with and without the cylindrical object. The modulus of the Fourier transform of the exit wave is replaced with the square root of the diffraction patterns without the cylindrical object at the even-numbered iterations and with the cylindrical object at the odd-numbered iterations. The object function is updated in each iteration. The probe functions are divided into several independent probe modes for the datasets both with and without the cylindrical object.

**Figure 5:**
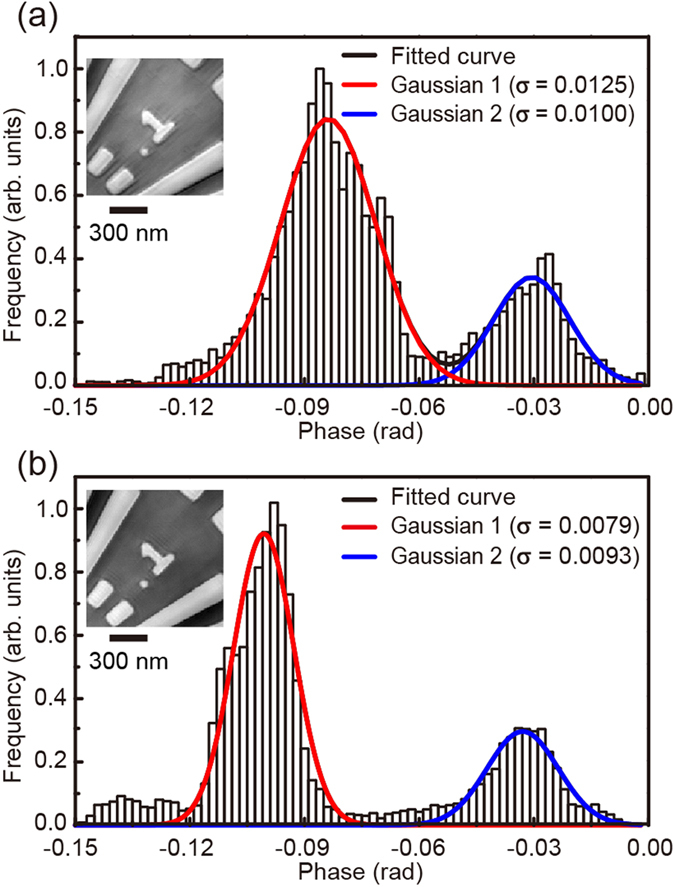
(**a,b**) Histograms of central 120 × 120 pixels of the reconstructed phase images in Fig. 3(b,c), which were fitted by a composite function comprising two Gaussian functions. (**a**) Dataset without the cylindrical object. (**b**) Both datasets with and without the cylindrical object.

We here propose a combined reconstruction algorithm for dark-field X-ray ptychography to reconstruct high-resolution images with few long-period artifacts, in which the reconstruction of the object employs two ptychographic datasets with and without the cylindrical object. Figure 4 shows a schematic diagram of the phase retrieval calculation. The detailed procedure is described in Methods. The square root of the diffraction patterns with and without the cylindrical object replaces the modulus of the Fourier transform of the exit wave at the even- and odd-numbered iterations, respectively. The object and the multiple probes are also updated at the even- and odd-numbered iterations, respectively. Figure 3(c) shows the image reconstructed using the combined algorithm. By comparison with the image in Fig. 3(c), it is clear that the long-period artifacts of the image are removed. To quantitatively evaluate the spatial resolution of the images, twelve line profiles for each image are analyzed. Figure 3(d) shows line profiles along the bold colored lines in Fig. 3(a–c). The FWHM was determined to be 33.7 ± 0.2 nm for the image in Fig. 3(a), 13.7 ± 0.1 nm for the image in Fig. 3(b), and 11.7 ± 0.1 nm for the image in Fig. 3(c). The average values for the images in Fig. 3(a–c) were 67.1, 13.0, and 11.5 nm, respectively. The present reconstruction algorithm results in the best resolution among these images. Figure 5(a,b) show histograms of the phase distribution in the selected areas of Fig. 3(b,c) inset in Fig. 5, respectively. The histograms were fitted by a composite function of two Gaussian functions. Here the phase resolution was obtained by measuring the standard deviation (σ) of the Gaussian fit. The σ values for the images in Fig. 3(b,c) are 0.0125 and 0.0079, respectively. The phase resolution was improved by removing the long-period artifacts. In addition, the interval between the peaks in Fig. 5(b) is 0.0677 rad, exactly corresponding to the theoretical phase for 30-nm-thick Ta at 6.5 keV. This means that the present coherent imaging technique is also a quantitative method.

### Multiple probe functions

**Figure 6:**
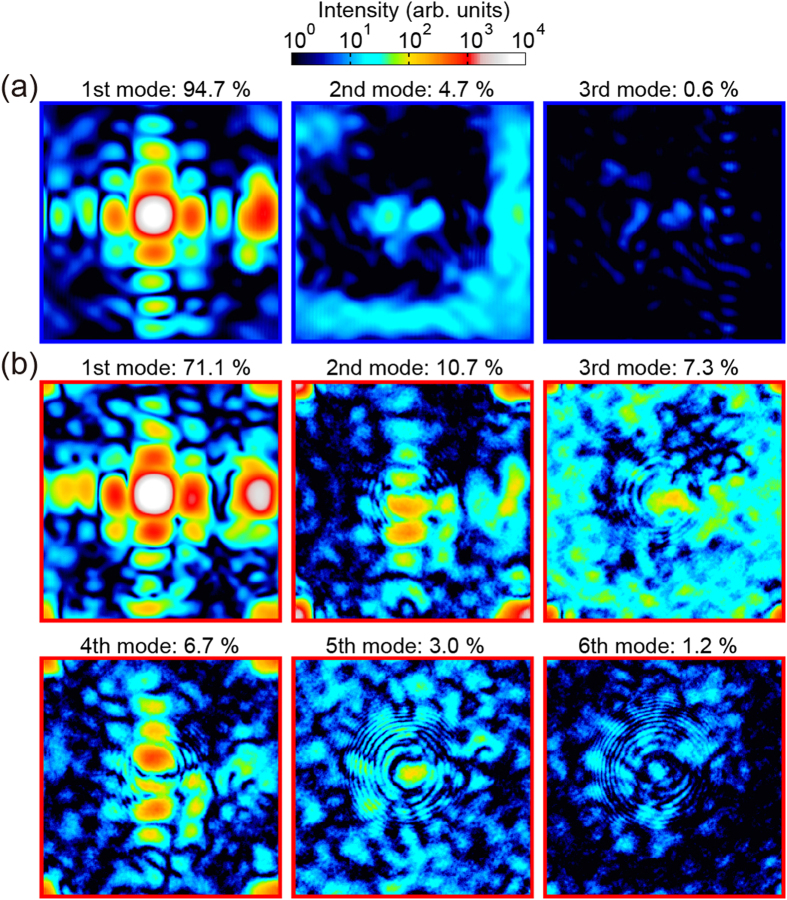
(**a,b**) Reconstructed probe modes (**a**) without and (**b**) with the cylindrical object. The population of each mode is given at the top of each figure.

Figure 6(a,b) show the intensity distributions of the reconstructed probe functions without and with the cylindrical object, respectively, after combined reconstruction. After the phase retrieval calculation, the Gram−Schmidt process was employed to orthogonalize the final probe modes, and then the population of each mode was derived. The first mode without the cylindrical object exhibits Fraunhofer diffraction from the rectangular aperture. In the second and third modes of the probe without the cylindrical object shown in Fig. 6(a), the peak divided into a few peaks, which was due to the poor spatial coherence of the synchrotron radiation in the horizontal direction^24^. Although the first mode of the probe with the cylindrical object is similar to that without the cylindrical object, its population decreases in spite of the secondary slit having the same opening.

This might be because the vibrations of the test pattern or the cylindrical object degrades the visibility of the diffraction patterns. If the cylindrical object simultaneously vibrates with the sample, vibrations should affect the higher order modes^25^. In the present measurement, the cylindrical object and the sample vibrate at different frequencies, which results in the appearance of additional coherent modes and the separation of the probe mode with the reference object. A pattern of concentric circles, which was generated from the cylindrical object, appears in the higher-order mode of the probe as shown in Fig. 6(b). Thus, the higher-order mode works as the reference wave for the in-line holography.

### Observation of magnetotactic bacteria, MO-1

**Figure 7:**
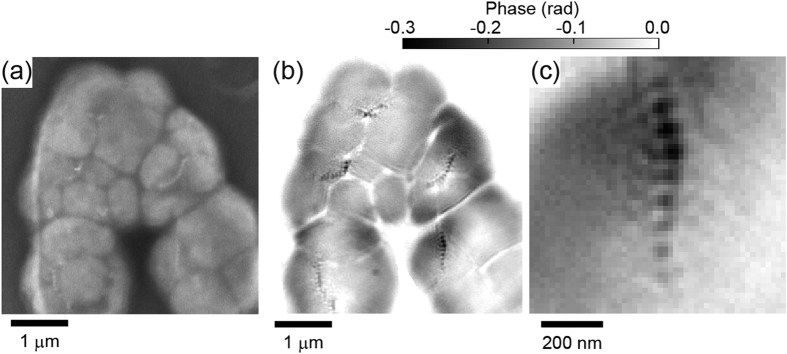
(**a**) SEM image of the magnetotactic bacteria MO-1. (**b**) Phase map of MO-1 obtained by dark-field X-ray ptychography. The pixel size is 17 nm. (**c**) Enlarged image of (**b**) showing the lower-right bacterium.

Dark-field X-ray ptychography was applied to the observation of the magnetotactic bacteria MO-1^26^. The sample preparation and experimental condition at SPring-8 are described in Methods. Figure 7(a,b) show an SEM image and the phase image reconstructed by dark-field X-ray ptychography in the same field of view, respectively. The pixel size of the reconstructed image is 17 nm. The sample consists of five bacteria. Each bacterium includes magnetosomes, which are chain structures of magnetite nanoparticles, inside the body. In the reconstructed image, magnetosomes are clearly visualized as black dots. Figure 7(c) shows an enlarged view of the magnetosomes of the lower-right bacterium. Each magnetic particle is resolved and seems to distribute between 20 and 70 nm, which is in good agreement with the TEM images of MO-1^27^,^28^. The phase image also provides information on the thickness of the sample. The theoretical densities of proteins and magnetite are 1.35 and 5.17 g/cm^3^, respectively. The thicknesses of the body and magnetite particles were estimated to be 300−400 and ~20 nm, respectively. The achieved spatial resolution of the protein in the X-ray imaging was estimated from the dose required for imaging, which was below the maximum tolerable dose^29^. In the present experiment, the absorbed doses at each scanning point without and with the cylindrical object were 1.3 × 10^9^ and 3.3 × 10^8^ Gy, respectively, where the former is larger than the dose required for 20 nm spatial resolution. The total dose, *i.e*., 1.63 × 10^9^ Gy, is close to the maximum tolerable dose. No degradation of the spatial resolution due to excessive radiation would occur.

## Discussion

We have modified dark-field X-ray ptychography and experimentally demonstrated it that allows us to realize the high-resolution projection imaging of a weak-phase test object. Although this method can be extended to the three-dimensional imaging by means of computed tomography (CT), further improvement of the apparatus is required since a long-term beam and thermal stability are needed for repeat measurements with/without a reference source. In addition, the measurement throughput must be dramatically improved. At present, many third-generation synchrotrons are undergoing or planning upgrades to be converted into low-emittance storage rings which provide high-flux coherent X-rays. It will be possible to perform the CT measurement of the dark-field X-ray ptychography by using the low-emittance synchrotron storage rings. A significant feature of dark-field X-ray ptychography is the compression of the dynamic range of intensities of diffraction patterns. The collection of diffraction patterns with a large dynamic range can now be achieved using photon-counting pixel detectors^30^,^31^,^32^. However, undetected photons will be a crucial issue for photon-counting pixel detectors when using the low-emittance sources. The maximum count ratio will limit the performance of X-ray ptychography. The use of a semitransparent central stop increases the effective dynamic range of the diffraction patterns^33^. This approach is very useful for strong-phase objects but not for weak-phase objects. A random hole array, which randomizes the phase structure of a probe, can reduce the dynamic range without reducing sensitivity^34^. However, it is difficult to reduce the dynamic range by more than one order of magnitude. Dark-field X-ray ptychography can overcome these limitations and provide ultimate resolution and sensitivity. We believe that the present method will open up a new frontier of X-ray visualization using next-generation synchrotron radiation.

## Methods

### Phase retrieval calculation

We provide the modifications to the real and reciprocal projections that allow for the ptychographic iterative engine to reconstruct a single object with the alternating multiple exit-wave functions 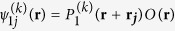 and 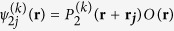, where 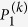 and 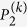 are the probe functions without and with the cylindrical object, respectively, and *O* is the object function. By rewriting the exit-wave function 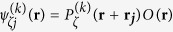 using the subscript


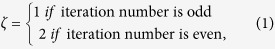


the multiple-mode ePIE^15^,^19^ equations can be written in the usual compacted form, starting from the following parameterization of the Fourier projecting constraint:


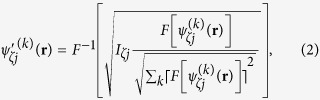


where *I*_*ξj*_ is the *j*_*th*_ diffraction pattern, *F* is the Fourier transform, and the summation proceeds over all *k* orthogonal modes. While the object can be in multiple states, the refinement of a static object is considered for simplicity only as


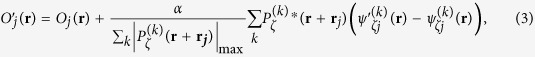


Thus, the modification of the probe refinement to account for multiple modes becomes trivial


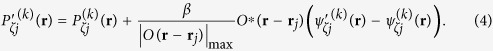


A loop over the *j*_*th*_ positions amounts to one iteration, whereas *n* iterations are needed to reach convergence.

### Sample preparation of magnetotactic bacteria MO-1

The magnetotactic bacteria MO-1 were cultivated in salt water. The culture solution was replaced by an aqueous solution of 0.5 mol/l acetic acid, which has the same osmotic pressure as the culture solution. The solution containing MO-1 was deposited on a 100-nm-thick SiN membrane and dried in air.

### Data acquisition of diffraction patterns of MO-1

The slit openings in the horizontal and vertical directions were 10 and 30 μm, respectively. The flux of the focused X-rays was estimated to be 9.0 × 10^8^ photons/s. The sample was illuminated in 11 × 11 overlapping fields of view that were separated by 400 nm in the horizontal and vertical directions. The exposure times at each position were 30 and 120 s for the measurements with and without the cylindrical object, respectively.

## Additional Information

**How to cite this article**: Suzuki, A. *et al*. Dark-field X-ray ptychography: Towards high-resolution imaging of thick and unstained biological specimens. *Sci. Rep*. **6**, 35060; doi: 10.1038/srep35060 (2016).
